# To the Survivors of the Medical Corps of the Confederate States Army

**Published:** 1890-05

**Authors:** Joseph Jones

**Affiliations:** Surgeon-General. United Confederate Veterans


Office of the Surgeon-General
Of the United Confederate Veterans,
156 Washington Avenue, New Orleans, La.,
April 9th, 1890.

*To the Survivors of the Medical Corps of the Confederate States Army:*

Comrades—The surrender of the Army of Northern Virginia on this day twenty-five years ago practically ended the struggle for the independence of the Southern States ; and during this quarter of a century death has thinned our ranks, and our corps can now oppose but a broken line in the great struggle against human suffering, disease and death. S. B. Moore, Surgeon-General of the Confederate Army, is dead ; Surgeons L. Guild, A. J. Foard, J. H. Berrien, J. T. Darby, W. A. Carrington, F. A. Ramsen, Samuel Choppin, R. J. Breckenridge, E. N. Coney, E. S. Gilliard, E. A. Flewellen, A. N. Tally, Paul F. Eve, O. F. Manson, Louis D. Ford, Habersham, James Bolton and a host of other medical officers of the Confederate States army are all dead.

The Association of the United Confederate Veterans was formed in New Orleans in 1888, the objects of which are historic, social and benevolent.

Our illustrious Commanding General, John B. Gordon, Governor of Georgia, has ordered the United Confederate Veterans to assemble in Chattanooga on July 2d, 1890. It is earnestly hoped that every surviving member of the Medical Corps of the Confederate army will meet with the United Confederate Veterans upon this important occasion, and promote by his presence and his counsels the sacred interests of the Association of the United Confederate Veterans.

It is of the greatest importance to the future historians, and also to the honor and welfare of the medical profession in the South, that careful records should be furnished the Surgeon-General of the United Confederate Veterans, embracing the following data:

1. Name, age, nativity, date of commission in the Confederate States army, nature and length of service of each and every member of the Medical Corps of the Confederate States army.

2. Obituary notices and records of all deceased members of the Medical Corps of the Confederate army.

3. The titles and copies of all field and hospital reports of the Medical Corps of the Confederate army.

4. Titles and copies of all published and unpublished reports relating to military surgery and diseases of armies, camps, hospitals and prisons.

The object proposed to be accomplished by the Surgeon-General of the United Confederate Veterans is the collection, classification, preservation and final publication of all the documents and facts bearing upon the history and labors of the Medical Corps of the Confederate States army during the civil war, 1861-65. Everything which relates to this critical period of our national history which shall illustrate the patriotic, self-sacrificing and scientific labors of the Medical Corps of the Confederate States army, and which shall vindicate the truth of history, should be industriously collected, filed and finally published.

It is believed that invaluable documents are scattered over the whole land, in the hands of the survivors of the civil war of 1861-65, which will form material for correct delineation of the medical history of the corps which played so important a part in the great historic drama.

Death is thinning our ranks, whilst time is laying its heavy hand upon the heads of those whose hair is already whitening with the advance of years and the burden of care. No delay, fellow-comrades, should be suffered in the collection and preservation of these precious documents. The task of collection of all documents, cases. facts relating to the medical history of the Confederate army, invites the immediate attention and co-operation of his honored comrades and beloved compatriots throughout the South. Respectfully, your obedient servant,

JOSEPH JONES, M. D.,
Surgeon-General United Confederate Veterans.

